# Improved prosthetic hand control with concurrent use of myoelectric and inertial measurements

**DOI:** 10.1186/s12984-017-0284-4

**Published:** 2017-07-11

**Authors:** Agamemnon Krasoulis, Iris Kyranou, Mustapha Suphi Erden, Kianoush Nazarpour, Sethu Vijayakumar

**Affiliations:** 1 Institute of Perception, Action and Behaviour, School of Informatics, University of Edinburgh, Edinburgh, UK; 2 Institute for Adaptive and Neural Computation, School of Informatics, University of Edinburgh, Edinburgh, UK; 3 School of Engineering and Physical Sciences, Heriot Watt University, Edinburgh, UK; 4 School of Electrical and Electronic Engineering, Newcastle University, Newcastle, UK; 5 Institute of Neuroscience, Newcastle University, Newcastle, UK

**Keywords:** Myoelectric prosthesis, Myoelectric control, Inertial measurement unit, Surface electromyography, Hand motion classification

## Abstract

**Background:**

Myoelectric pattern recognition systems can decode movement intention to drive upper-limb prostheses. Despite recent advances in academic research, the commercial adoption of such systems remains low. This limitation is mainly due to the lack of classification robustness and a simultaneous requirement for a large number of electromyogram (EMG) electrodes. We propose to address these two issues by using a multi-modal approach which combines surface electromyography (sEMG) with inertial measurements (IMs) and an appropriate training data collection paradigm. We demonstrate that this can significantly improve classification performance as compared to conventional techniques exclusively based on sEMG signals.

**Methods:**

We collected and analyzed a large dataset comprising recordings with 20 able-bodied and two amputee participants executing 40 movements. Additionally, we conducted a novel real-time prosthetic hand control experiment with 11 able-bodied subjects and an amputee by using a state-of-the-art commercial prosthetic hand. A systematic performance comparison was carried out to investigate the potential benefit of incorporating IMs in prosthetic hand control.

**Results:**

The inclusion of IM data improved performance significantly, by increasing classification accuracy (CA) in the offline analysis and improving completion rates (CRs) in the real-time experiment. Our findings were consistent across able-bodied and amputee subjects. Integrating the sEMG electrodes and IM sensors within a single sensor package enabled us to achieve high-level performance by using on average 4-6 sensors.

**Conclusions:**

The results from our experiments suggest that IMs can form an excellent complimentary source signal for upper-limb myoelectric prostheses. We trust that multi-modal control solutions have the potential of improving the usability of upper-extremity prostheses in real-life applications.

**Electronic supplementary material:**

The online version of this article (doi:10.1186/s12984-017-0284-4) contains supplementary material, which is available to authorized users.

## Background

Upper-limb myoelectric prostheses aim at replacing the appearance and functionality of a missing limb. In academic research, pattern recognition-based systems have been very successful in decoding movement intent and have recently found their way into commercial products^1^. Classification methods have been extensively used for decoding grip type, wrist and individuated finger movement [1–6]. Moreover, the real-time performance of such systems has been evaluated with able-bodied and amputee subjects [7–9]. Nevertheless, the acceptance of myoelectric systems by end users has been remarkably low [10]. This is mainly due to limited classification robustness which can be partially improved by increasing the number of used electromyogram (EMG) sensors. The use of a large amount of sensors, however, may be impractical for the user. To further improve the clinical adoption of upper-limb prostheses, next generation myoelectric systems will have to exhibit multi-modal control and use a minimal amount of sensors [11].

The sEMG signal is inherently noisy and thus, not a robust source of input information for prosthetic systems [12]. This is especially true for altered conditions such as sweat, fatigue, and electrode displacement [11]. Therefore, it is imperative to move towards multi-modal control solutions.

One of the main issues associated with the use of the sEMG signal is the *limb position effect* which states that a system trained on a single arm position is likely to fail to generalise to different arm postures [13]. Fougner et al. [14] proposed to address this issue by training decoders in multiple limb positions and also by using accelerometers placed on the forearm and the biceps muscle of the subjects to measure arm orientation. Their approach resulted in a substantial decrease in classification error from 18 to 5%. Geng et al. [15] later reproduced this finding in amputee subjects by training a two-stage (position/motion) classifier. Radmand et al. [16] demonstrated, however, that myoelectric decoding can benefit from the use of accelerometers only if training data are collected across many positions, which may be infeasible in practice. To overcome this issue, they proposed to collect training data with dynamic movements during which the (residual) arm is moved through the regions of interest. Khushaba et al. [17] investigated the combined effect of forearm orientation and muscular contraction level and verified that the use of accelerometers can be beneficial for classification performance. Finally, the combination of (EMG) and accelerometry has been also found to improve classification accuracy (CA) in lower-limb movement intent decoding [18]. Nevertheless, all the aforementioned studies were limited to offline analyses. Yet, it is unclear whether the observed increase in offline CA can be associated with a performance improvement during real-time, task-oriented myoelectric control [19].

The goal of the current study has been threefold: 1) to investigate whether classification performance can further benefit from the use of additional inertial sensors, such as gyroscopes and magnetometers; 2) to assess whether an increase in offline CA can be translated to a performance improvement during real-time prosthetic control; 3) to investigate whether the inclusion of inertial measurements (IMs) can help reduce the number of sensors required to achieve robust classification performance. This last aspect is particularly important for real-life applications, where it is desirable to minimise the number of sensors used by the prosthesis.

## Methods

This study comprised two sets of experiments. In the first part, we recorded data from 22 subjects (20 able-bodied, two amputees). A systematic offline analysis was then performed on decoding hand movement from myoelectric and IM signals. Based on observations from our offline analysis, we proceeded with the second part of the study which involved carrying out a real-time, prosthetic hand control experiment with 12 subjects (11 able-bodied, one amputee). A summary of the two sets of experiments is given in Table 1. The medical records of the amputee participants are provided in Table 2.

**Table 1:** Experiments summary

Experiment	Able-bodied	Amputee	Number of	Decoding
	subjects	subjects	classes	conditions
Offline	20	2	40	8
Real-time	11	1	6	4

**Table 2:** Amputee subjects medical records

Gender	Age	Type of amputation	Cause of amputation	Years	Missing limb	Hand dominance (prior to amputation)	Prosthesis use	Experiment
Male	28	Transradial	Car accident	6	Right	Right	Split hook	Offline /Real-time
Male	54	Transradial	Cancer (epitheliod sarcoma)	18	Right	Right	Split hook	Offline

Identical procedures were followed in the two sets of experiments for signal acquisition, conditioning (i.e. preprocessing and feature extraction), and motion/grasp classification. The main differences were with regards to the performed tasks and the metrics used to evaluate performance.

### Signal acquisition

**Fig. 1:**
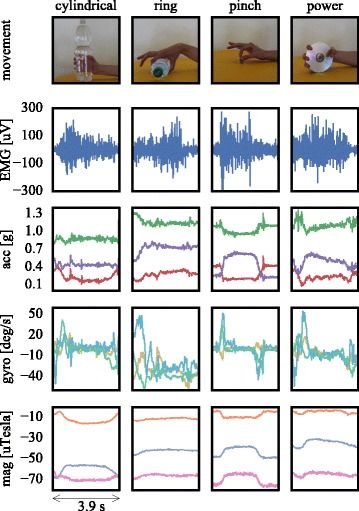
Raw sEMG and IM data (offline experiment). Traces of raw signals associated with a single EMG-IM sensor are shown for four movements (top panel). The IMU components comprised tri-axial accelerometers (acc), gyroscopes (gyro), and magnetometers (mag) measuring 3D acceleration, angular velocity, and magnetic field, respectively (bottom three panels). Photographs showing movements (top panel) have been reproduced from [20] and are licensed under a Creative Commons Attribution 4.0 International License (https://creativecommons.org/licenses/by/4.0/)

Myoelectric and IM data were collected by using a Delsys^®^ Trigno^TM^ IM Wireless System^2^. Each EMG electrode incorporated a 9-degree-of-freedom (DOF) IMU, that is, a tri-axial accelerometer, gyroscope and magnetometer measuring acceleration, angular velocity and magnetic field, respectively. Therefore, the number of raw signals associated with each EMG-IM sensor was 10 (each column in Fig. 1).

The sampling frequency was set to 2 kHz for myoelectric signals and 128 Hz for IM data. Since IM readings were used in their raw format, no calibration was required.

**Fig. 2:**
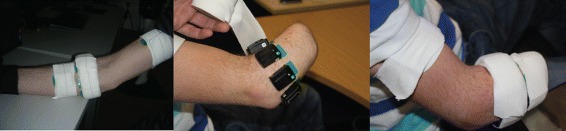
Sensor placement. Eight EMG-IM sensors were equally spaced around the participants’ forearm (3 cm below the elbow), two targeted the EDC and FDS muscles, and two were placed on the biceps and triceps muscles. Elastic bandage was used to keep sensors positions fixed. Sensor placement shown for an able-bodied (*left*) and an amputee subject (*centre*, *right*)

For sensor placement, we followed the NinaPro protocol [20] and used 12 sensors. Eight sensors were equally spaced around the forearm (placed 3 cm below the elbow), two targeted the extrinsic hand muscles (extensor digitorum communis (EDC), digitorum superficialis (FDS)), and the remaining two were placed on the biceps and triceps brachii muscles. Prior to electrode placement, participants’ skin was cleansed by using 70% isopropyl alcohol. Adhesive latex-free elastic bandage was used to keep the positions of the sensors fixed throughout the experimental sessions. Representative pictures showing electrode placement for two participants (one able-bodied and one amputee) are shown in Fig. 2. A summary is also provided in Table 3.

**Table 3:** EMG-IM sensor placement

Sensor	Location
1-8	Equally spaced around forearm (3 cm below elbow)
9	Targeting EDC
10	Targeting FDS
11	Biceps brachii
12	Triceps brachii

### Offline data collection

Data were collected offline with 20 able-bodied and two amputee subjects by adopting the NinaPro protocol [20, 21]. Subjects were asked to reproduce a series of 40 motions, including various individuated-finger, hand, wrist, grasping and functional movements (exercises B and C in [21]) instructed to them on a computer screen. Each movement was repeated six times and trials were interleaved with 5-s resting periods. The two amputee volunteers were instructed to perform bilateral imaginary mirrored movements^3^.

Following Gijsberts et al. [22], power line interference was suppressed from the myoelectric signals by applying a Hampel filter. The post-hoc relabeling procedure that was described in the same study was used to identify and refine the exact stimuli timings for each subject and trial in order to avoid introducing label-related noise in the classifiers. The cause of this type of noise is the natural variability introduced when subjects replicate movements instructed to them on a screen (i.e. onset delays, variability in trial lengths, etc.).

### Real-time control pick and place experiment

**Fig. 3:**
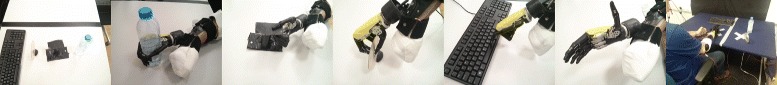
Real-time control pick and place experiment. Participants were instructed to use a prosthetic hand to grasp and relocate three objects and finally press the ‘space’ key on a computer keyboard (*left*). Five grip types were used: power/cylindrical (water bottle), lateral (credit card simulator), tripod (CD), index finger pointer (computer keyboard) and hand open. A trial with an amputee participant is shown on the *right*

For the real-time experiment, all subjects were fitted the Touch Bionics®; robo-limb™ prosthetic hand^4^ on their right arm by using a custom-made socket. The robotic hand was controlled in real-time by using EMG and/or IM data as input(s). The experimental task, objects used and associated hand grips are presented in Table 4 and Fig. 3. Participants were instructed to use the hand to grasp, lift and relocate a series of objects and finally press the ‘space’ button on a computer keyboard. Three objects were used and participants were required to lift each object with an associated grip type which was instructed to them. In total there were six classes, including the hand ‘open’ and ‘rest’ (i.e. no action taken) poses. The fingers of the able-bodied participants’ right hand were constrained in a fist formation throughout the experimental sessions by using elastic bandage in an effort to mimic limb loss as closely as possible.

**Table 4:** Real-time experiment objects used and associated grip types

Class	Object	Grip
0	-	Rest pose
1	Bottle	Power
2	Credit card simulator	Lateral
3	Compact Disc (CD)	Tripod
4	Keyboard key	Index pointer
5	-	Open pose

Each session comprised a training and a testing phase. During the *training* phase, participants were required to perform five reach-to-grasp repetitions of each of the five poses/grips (classes 1-5 in Table 4). Throughout this stage, participants were instructed to move their arm at a steady pace and activate their muscles in a natural way, that is, without exerting excessive tension. The objects corresponding to the different poses were placed on a computer desk in front of the participants, however they were not able to physically grasp them, due to their fingers being constrained by the elastic bandage. During this phase, which was required to collect training data, the prosthetic hand was kept inactive. To indicate the motion being performed, participants were asked to press down with their contralateral hand a corresponding key on a computer keyboard, with a different key corresponding to each of the performed poses (i.e. 1-5). The amputee participant performed ten repetitions of each movement.

In the interval between the training and testing phases, participants were given a 5-min break. During this time, four different classifiers were trained. The classification schemes corresponded to the following four conditions, according to the source(s) of input data that were used for decoding (Table 5):

I. sEMG data from all sensors.

**Table 5:** Real-time experiment decoding conditions

Condition	Input	Number of sensors	Input feature
			dimensionality
I	sEMG	12	84
II	IM	12	108
III	sEMG-IM	12	192
IV	sEMG-IM (subset)	3-7	48-112
II. IM data from all sensors.
III. sEMG and IM data from all sensors.
IV. sEMG and IM data from a selected subset of sensors.

During the *testing* phase, each trial consisted of picking and placing the three objects approximately 50 cm away from their initial position. A trial ended by pressing the space button on the computer keyboard by using the index pointer grip. Able-bodied subjects were given 60 s to accomplish the trials with the prosthetic hand and the amputee participant was given 75 s. The objects were presented to the subjects in a pseudo-randomised order, so that the sequence of required grasping motions varied across trials. Able-bodied subjects performed four trials for each of the four decoding conditions and the amputee participant performed six. When the prosthetic hand performed a different movement than the one intended by the user, for instance due to a motion misclassification, participants were asked to open the hand and try performing the intended movement again. The presentation order of the four decoders described above was counterbalanced across the able-bodied population in order to avoid favouring certain conditions over others, given the learning mechanisms taking place during prosthetic control [2, 19, 23]. The total duration of experiments including skin preparation, sensor placement, training, and testing was around 90 min for each subject.

A finite-state machine implementation was used for the real-time control of the prosthetic hand. A movement predicted by the classifier was triggered only if the most recently performed movement had terminated execution. To determine movement execution termination, the hand’s motor current readings were constantly monitored and compared to a fixed threshold. In addition, a control command was triggered only when it was predicted with high confidence, in other words when the posterior probability of the corresponding class exceeded a threshold. The probability threshold was set a priori to *θ*=0.995. For the real-time experiment, signal acquisition, pre-processing and control of the prosthetic hand were implemented in C++ and integrated into the Robot Operating System (ROS) [24]. The communication between ROS and the hand was achieved via the CANBUS protocol.

### Signal preprocessing and classifier training

Myoelectric and IM signals were synchronised via linear interpolation. By using a shifting window approach, four sEMG features were extracted from each channel, namely the mean absolute value (MAV), waveform length (WL), 4th-order auto-regressive (AR) coefficients and log-variance (LogVar). The selection of these features was based on previous studies demonstrating their efficacy in decoding hand motion intention [25–27]. Bearing in mind the need for low computational requirements during real-time control, we only considered time-domain (TD) sEMG features [28]. The length of the shifting window was set to 256 ms and the increment to 50 ms (80% overlap). It has been previously shown that this selection offers a good compromise between classification performance and controller delay [29]. For the real-time experiment, the average delay was 170 ms [30] which is within the acceptable range for the purposes of upper-limb myoelectric control [31]. In order to match EMG features, IM data were also binned in 256 ms windows by extracting the mean value (MV) of the signals within the processing window. The total number of features contributed by each sensor was thus 16 (7 EMG, 9 IM features.)

The columns of the design matrix (i.e. input features) were standardised by subtracting the mean and dividing by standard deviation. For the offline experiment, mean subtraction and feature scaling followed cross-validation splitting and were thus performed by using training data only. For the real-time experiment, mean and standard deviation vectors for each subject were estimated on the entire training dataset and subsequently used to transform the input data during the testing phase. Training datasets collected for both types of experiments were included unchanged in the subsequent analyses steps, in other words, no segments of activity were manually removed.

For movement intent decoding from myoelectric and IM data, we employed a linear discriminant analysis (LDA) classifier. Discriminant analysis is a family of supervised dimensionality reduction algorithms for identifying feature projections that maximise class separability. These methods can be used for multi-class classification by assuming a class-conditional Gaussian model. The linear discriminant analysis (LDA) is a special case of this family which assumes a shared covariance matrix across the different classes resulting in linear decision boundaries (i.e. hyperplanes). In the context of myoelectric control, LDA and its variants have been extensively used, since they can achieve high decoding performance with minimal requirements [3–5, 32].

One particular advantage of discriminant analysis classifiers is that they are probabilistic, that is, they estimate a posterior probability distribution over classes as opposed, for instance, to support vector machines (SVMs) which only yield a classification decision. This feature was particularly important for our paradigm where confidence-based classification rejection was deployed at the final control stage. Another strong advantage of LDA is its efficiency at test time; both time and space complexities scale linearly with the input feature dimensionality [33].

For both experiments, the extracted sEMG and/or IM features were fed as input(s) to the classifiers and the vectors containing the stimulus time-series (i.e. grip performed) were used as targets. All four types of classifiers (Table 5) were trained and tested by using data from individual subjects.

### Decoding performance assessment

Decoding performance was evaluated differently in the two experiments. For the offline experiment, five repetitions were used to train the decoders and the left-out repetition was used to assess decoding performance. The procedure was then repeated by using a different evaluation fold in each iteration, hence resulting in a 6-fold cross-validation. Following classification, the class distribution of the test folds was balanced by removing a large proportion of the instances corresponding to the rest class. This step was necessary to prevent CA scores from being biased by the aforementioned class. The identity of rest samples to be removed was determined according to their temporal distance from the nearest segment of muscle activity and, hence, the repeatability of our analysis was not affected by this procedure.

Finally, to evaluate decoding performance we used the standard classification accuracy (CA) metric defined as:

1 $$ {\text{CA}} = \frac{\text{correctly classified instances}}{\text{total classified instances}} \times 100\%.  $$

For the real-time experiment, we adopted two task-related metrics which are commonly used in the literature [8, 9]. The completion rate (CR) is defined as the ratio of successful to total number of trials, and completion time (CT) is defined as the time taken to complete a successful trial. A trial was considered successful only if it was accomplished within a given time frame (60 s for the able-bodied subjects and 75 s for the amputee participant).

### Sequential forward sensor selection (SFSS)

One of the main aims of this study was to assess whether the use of inertial data measured with the same sensor packs that record EMG signals could help reduce the number of channels required to achieve high-level myoelectric control. Therefore, we investigated whether the use of an optimally selected subset of EMG-IM sensors could achieve the same level of decoding performance attained by the decoders when all available sensors were used.

A sensor selection method was developed which was based on the classic sequential forward feature selection algorithm [1, 34, 35]. Our adapted algorithm was initialised with an empty sensor set. In each iteration, the sensor which yielded the highest performance improvement was added to the pool. Decoding performance was assessed by including all input signals from the associated sensor, in other words 7 sEMG and 9 IM features. To increase the robustness of our method, cross-validation was used in each iteration and the sensor selection decision was based on a majority vote across the cross-validation folds. For consistency, the CA metric was used for assessing decoding performance in each step. The algorithm terminated execution once all sensors were included in the set, in other words when all available sensors were ranked according to their relative predictive power. For both the offline and real-time experiments we selected those sensors the addition of which yielded an improvement in CA larger than 1%. The sensor rankings varied across subjects, therefore a different subset was used for each subject. The size of the subset also varied across subjects. For the real-time experiment, sensor selection was performed by using the training data only and the sensor subset for each participant was kept fixed throughout the testing phase.

## Results

### Offline analysis

**Fig. 4:**
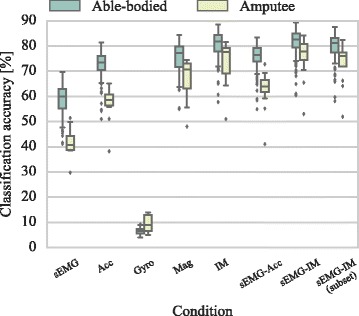
Offline experiment decoding performance comparison. Balanced classification accuracies shown for the able-bodied and amputee populations. Data shown for all subjects (20 able-bodied, two amputees) and cross-validation folds (*k*=6). Straight lines, medians; solid boxes, interquartile ranges; whiskers, overall ranges of non-outlier data; diamonds indicate outliers. sEMG, surface electromyography; Acc, accelerometer; Gyro, gyroscope; Mag, magnetometer; IM, inertial measurements (accelerometer, gyroscope, magnetometer)

Our first aim was to assess the predictive performance of the different modalities explored in this study, that is, the sEMG signal, accelerometer, gyroscope, magnetometer data, and various combinations thereof. A systematic comparison was performed on the balanced CA achieved by various decoders on a large pool of gestures and hand movements (40 classes). We also examined the case of including both EMG and IM information from an optimally selected subset of sensors. The results for both the able-bodied and amputee populations are presented in Fig. 4.

A Friedman test was performed to assess the effect of decoding condition on CA scores. A statistical significant effect was identified (*p*<10^−3^) and pair-wise comparisons were subsequently performed by using the Nemenyi test. For both populations, the performance of the sEMG-IM classifier was significantly higher than that of any other decoder (median balanced CA for this condition was 82.7% for able-bodied and 77.8% for amputee subjects). This is not surprising because this was the condition that all available sensorial information was used for classification. The second best performance was achieved by the IM decoder (81.7% able-bodied, 77.7% amputees), followed by the sEMG-IM subset condition (81.2% able-bodied, 76% amputees) although the differences between the latter two conditions were not statistically significant.

One of the motivations of this study was to identify whether the additional inclusion of gyroscope and magnetometer data beyond accelerometry would be beneficial for hand movement decoding. Our offline analysis provided evidence supporting this hypothesis, since it was found the sEMG-IM decoder performed significantly better than sEMG-accelerometer. That was also the case when we completely discarded myoelectric data, in other words the IM decoder significantly outperformed the accelerometer classifier. Importantly, all comparisons were consistent across the able-bodied and amputee populations.

**Fig. 5:**
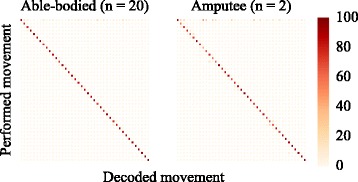
Offline experiment classification. Confusion matrices shown for the able-bodied (*left*) and amputee (*right*) populations for the sEMG-IM decoder. Results were averaged across participants (20 able-bodied, 2 amputees) and cross-validation folds (*k*=6). Colour intensities indicate normalised prediction scores for each class

Average confusion matrices for the best-performing condition (sEMG-IM, all sensors) are shown in Fig. 5 separately for the able-bodied and amputee groups. Confusion matrices for individual subjects were visually inspected and found to be similar to those shown in Fig. 5.

### Real-time control experiment

**Fig. 6:**
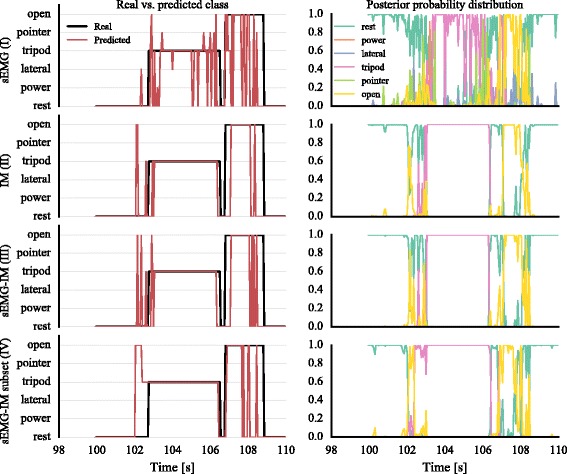
Real-time experiment classification. The real and predicted classes with the four different decoders are shown on the *left column*. The evolution of the posterior probability distribution for each classifier is shown on the *right column*. Representative traces shown for one subject by using training data and 3-fold cross-validation. sEMG, surface electromyography; IM, inertial measurements

Next, we turn our attention to the results of the real-time experiment described in the previous section. The working principle of the real-time classification system is illustrated in Fig. 6. The time series for the real and predicted classes with each of the tested classifiers (Table 5) are shown on the left column of the graph. The temporal evolution of the posterior probability distribution for each classifier is also shown in the same figure (right column). Evidently, for this segment of activity, the inclusion of IM data increased the robustness of the classifier. For the subject used in this example, six sensors were used in condition IV (sEMG-IM subset).

**Fig. 7:**
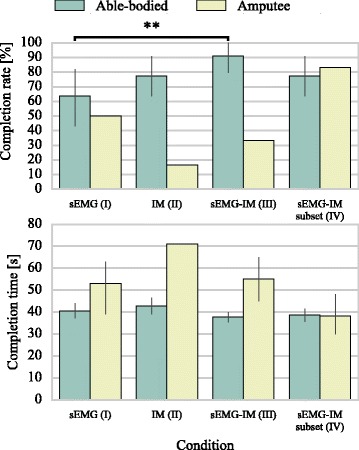
Real-time experiment decoding performance comparison. Average completion rates and times presented for four decoding conditions. Data shown for all subjects (11 able-bodied, one amputee) and trials. Vertical lines represent 95% confidence intervals estimated via bootstrapping (100 iterations). sEMG, surface electromyography; IM, inertial measurements; ^**^, *p*<10^−2^

Performance results for the real-time control experiment are summarised in Fig. 7. Analogous to our offline analysis, for the able-bodied group, the highest average CR was achieved with condition III (sEMG-IM classifier). The average CR in that case was significantly higher than for condition I, that is, when solely sEMG information was used (*p*<10^−2^, Cochran’s Q test followed by post-hoc pair-wise tests by using Bonferroni correction for multiple comparisons). The observed pattern was consistent across 10 out of the 11 able-bodied participants. No significant differences were identified among conditions I, II, and IV, although CRs for II and IV were on average 13-14% higher than that for condition I.

In terms of CTs, the performance of the four conditions was comparable. Nevertheless, condition III achieved marginally better results (i.e. lower mean CT) than the other three conditions.

For the amputee participant, we observed a slightly different pattern. The best decoding performance both in terms of CR and average CT was achieved with condition IV, when sEMG and IM data were used from a subset of sensors. Three sensors were used in this experiment, one of which targeted the FDS muscle, whilst the other two captured the activity of the extensor muscle group (sensors 1, 2 and 10 in Table 3).

The error bars in Fig. 7 represent 95% confidence intervals estimated via bootstrapping (100 iterations). Since there was only one amputee participant in this experiment, there was a single sample for CR (defined as the fraction of successful to total number of trials), thus no confidence interval was estimated for this measure. Similarly, for condition II, there was only one successful trial, therefore no confidence interval was estimated for the associated CT.

**Fig. 8:**
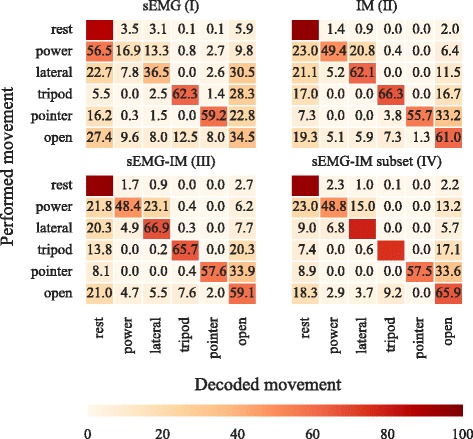
Representative confusion matrices for real-time experiment. Predictions shown for one able-bodied subject and four decoding conditions. *Colour bar* and *annotated scores* represent normalised prediction rates. Confusion matrices have been computed by using training data and 3-fold cross-validation. sEMG, surface electromyography; IM, inertial measurements

Representative confusion matrices for the real-time experiment are shown in Fig. 8. These correspond to one subject and all four decoding conditions. Inspection of the confusion matrices suggests that for this particular subject inclusion of IM data helped disambiguating the ‘power’ from ‘rest’, and the ‘lateral’ from ‘open’ classes. To estimate these confusion matrices training data were used by applying 3-fold cross-validation. Estimating confusion matrices during the testing phase of the real-time experiment would not be possible, since the ground truth, in other words, the participant’s intention is not known. This is mainly due to the sequential nature of the trials; within a single trial subjects were required to produce a series of motions, the exact timings of which are neither known, nor can be inferred by the experimenter.

**Fig. 9:**
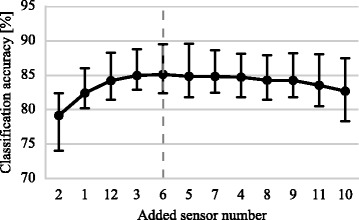
Sequential forward sensor selection (SFSS) example for one able-bodied subject (real-time experiment). The cross-validated classification accuracy is shown as sensors are added to the pool. The *dashed line* represents the termination of the sensor selection process as further inclusion does not yield an improvement in classification performance. *Error bars* represent 95% confidence intervals estimated via bootstrapping (1000 iterations)

**Fig. 10:**
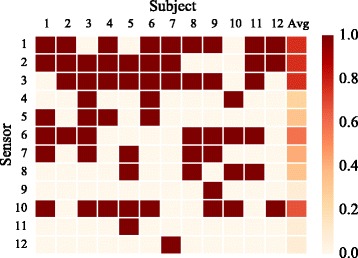
Sensor selection for individual subjects (real-time experiment). The selected EMG-IM sensors are shown column-wise as *red boxes* for 11 able-bodied and the amputee (column 12) subjects. The right-most column represents the average selection frequency for individual sensors. The reader is referred to Table 3 and Fig. 2 for details on sensor placement

A typical example of the SFSS procedure for selecting the subset used in condition IV is shown in Fig. 9. The selection of EMG-IM sensors for all participants in the real-time experiment is presented in Fig. 10. The number of selected sensors varied from 3 to 7 although it was typically in the range of 4 to 6 (10 out of 12 subjects). The average selection frequency of individual sensors is also shown in the same graph (right-most column).

### Reconstruction of EMG envelopes with IM

**Fig. 11:**
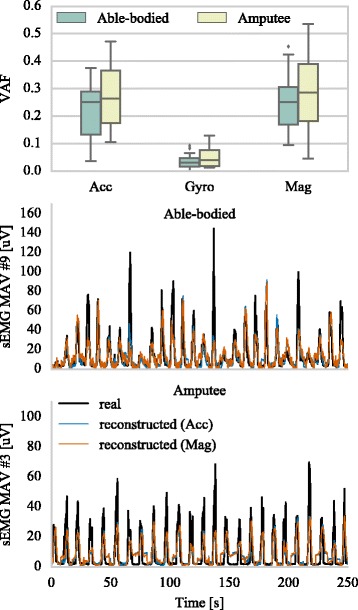
Electromyogram reconstruction from IMs. The sEMG variance accounted for (VAF) by accelerometer-, gyroscope- and magnetometer-based linear regression models is shown in the *top* panel. Reconstruction examples of sEMG signals from accelerometer and magnetometer data are shown in the *middle* (able-bodied) and *bottom* panels (amputee)

Finally, we sought to explore the relationship between the EMG and IM measurements. Concretely, we tried to identify the degree to which the different types of IM data might be related to the EMG measurements. In that direction, we built simple linear regression models to reconstruct the envelopes (i.e. MAV feature) of the sEMG signals from accelerometer, gyroscope, and magnetometer measurements, respectively. This process was performed individually for each sensor, that is, the reconstruction of each sEMG signal was achieved by using accelerometer, gyroscope, or magnetometer data from the same sensor only. The results of this experiment are shown in Fig. 11. The accelerometer and magnetometer data were able to capture on average 25-30% of the variance of the sEMG envelopes. Conversely, it was not possible to decode EMG activity by using gyroscope data. Examples of sEMG envelope reconstruction with accelerometer and magnetometer measurements are shown in the same graph, both for able-bodied and amputee subjects.

## Discussion

In this study, we investigated whether the performance of myoelectric decoders can benefit from the inclusion of additional information as measured by IMUs integrated within the EMG sensors. For this purpose, we collected a large dataset of 22 subjects performing a variety of movements and also conducted a real-time control pick and place experiment. The main contribution of this work has been threefold; including information from additional inertial sensors beyond accelerometers, minimizing the amount of sensors used for decoding and, most importantly, validating findings during real-time myoelectric control.

For our first (offline) experiment, the large number of classes makes gesture recognition a challenging task. We found that by including IM data, the CA increased by a significant factor. For the able-bodied group, we achieved a median CA of 82.7%, observing an increase in performance of 22.6% when compared to the sEMG-only case. For the amputee group, the same measure was 77.8% and the observed increase in performance was 37.1%. Remarkably, CA for the amputee group almost doubled when we included IM data in our decoders. To the best of our knowledge, this score is the highest ever reported for amputee subjects, given the large number of motions (i.e. 40 classes) in the dataset. For comparison, Atzori et al. [20] reported an average CA of 48% for the same set of movements. Other studies have achieved CAs as high as 96.6%, however for much smaller numbers of motions (i.e. 12 classes) [6].

Many studies have suggested that an observed increase in CA attained with purely offline analysis does not necessarily translate to performance improvement during online myoelectric control [19, 36]. Therefore, to validate our findings from the offline analysis, we conducted a real-time experiment by controlling a state-of-the-art commercial prosthetic hand. Comparing the real-time performance of all decoding schemes explored in our offline analysis would be impossible due to time constraints. By taking into account the results from the offline analysis, we decided to test the real-time performance of the four conditions presented in Table 5. Moreover, it is not practical to include 40 classes in a real-time control experiment, and perhaps not necessary from a clinical point of view. Therefore, we only included six classes (Table 4, Fig. 3) which have been previously identified as being the most useful from a user’s perspective [37]. It is worth noting that our proposed experimental protocol bears strong similarities to the “object task” of the Southampton hand assessment procedure (SHAP) test^5^ that is commonly used in clinical environments.

In comparison with similar studies which previously employed the (TAC) test [7–9, 38], our experimental task was more challenging. Participants were required to trigger a sequence of control signals (seven in total including the required intermediate ‘open’ commands), rather than performing a single grasp motion. Additionally, participants were given a rather short time to accomplish trials (60 s for the able-bodied group, 75 s for the amputee subject). We considered this paradigm as a more realistic experiment that closely matches real-life applications.

Radmand et al. [16] demonstrated that integrating accelerometry data into myoelectric decoders can potentially decrease decoding performance unless training data are collected under most of the possible configurations in 3D space. They also showed that classifiers trained with static motions generalise poorly when used to decode hand gestures during dynamic movement. To overcome this limitation, and since collecting static training data in all possible positions would be practically impossible, they proposed a method for training classifiers with dynamic movements covering the regions of interest. Since our offline experiment involved static hand motions, we considered imperative to further validate our findings during real-time prosthetic control. During the training phase of the real-time experiment, participants were instructed to move their arms within a constrained workspace (60 cm × 50 cm × 30 cm) whilst performing the different grips. This was inspired by the work of Radmand et al. [16]. Although this approach helped disambiguating muscle activity patterns under different postures, its potential to generalise to postures not present in the training set remains to be investigated. For instance, in our experiments training and testing used the same arm postures for same grip types, which is likely to favour the sEMG-IM decoders, since IM signals are posture dependent. Future work should test the trade-off between the benefit of using IM signals and generalisability under novel postures, i.e. by mixing grip types and arm postures. For clinical applications, acquisition of large and versatile datasets may be required to capture arm posture-related variability, and thereby ensure classification robustness.

Results from the real-time experiment were mostly in accordance with observations from the precedent offline analysis. We found that the inclusion of IM information resulted in significant improvement in CRs for the able-bodied group (median increase of 25%). One notable difference was that while offline analysis suggested that the use of IM data alone could achieve comparable CA to sEMG-IM classifiers (0.9% median difference), in our real-time experiment the hybrid decoders outperformed, although not significantly, IM classifiers (75.0% and 100.0% median CRs; 48.0 and 37.5 s median CTs for conditions (II) and (III), respectively). Such discrepancies between offline CA scores and task-related metrics have been previously reported in other studies [19, 36]. It has been commonly accepted that the latter should be regarded as more important than the former, since task-related metrics measuring the performance of real-time prosthetic systems are of greater clinical relevance than offline accuracy [39].

The trend observed for the amputee participant was slightly different to the one corresponding to the able-bodied pool. We seek to provide the following justification for this discrepancy: amputations are different and, consequently, skin conditions and positioning on the muscles can vary significantly. Therefore, there is a need to adopt a personalised approach for trans-radial amputees [3]. Although we observed that for the specific amputee participant the IM decoder (condition II) did not achieve as high performance as the sEMG decoder (condition I), we found that the highest performance was achieved when all modalities were included (condition IV). This provides further support for our proposal for sensor fusion, as we believe that by including additional modalities it is more likely to capture a richer representation of the underlying muscular activity.

The best performance for the amputee participant both in terms of CRs and CTs was achieved when we combined sEMG and IM data but made use of a smaller subset of the available sensors. The performance was inferior when the whole set of sensors was used. One possible explanation for this observation is that the participant was able to develop a more efficient control strategy in the former case, due to the lower dimensionality of the input space [40]. Nevertheless, the chance of observing a statistical error in this case, due to the small sample size, cannot be neglected.

A previous study reported high offline CA by discarding the EMG signal and using solely acceleration signals [22]. We were able to replicate this finding (Fig. 4), and we additionally found that a high CA could also be achieved by using magnetometer data only. Importantly, we further demonstrated that efficient real-time control was feasible by using exclusively IM data (Fig. 7). It is worth noting, however, that CTs were slightly increased for this condition. The first commercial system using IM data as sensory input has recently appeared on the market^4^, although its working principle is fundamentally different. To the best of our knowledge, our study is the first to demonstrate that real-time prosthetic control can be achieved by using a biomimetic approach and IM data exclusively. This finding cannot be solely attributed to a potential association of arm postures to grips since in our experiments participants mainly employed two arm postures, each of them associated with two different grips; for the “cylindrical” and “lateral” classes, the palm of the prosthesis was required to be perpendicular to the surface, while for the “tripod” and “index pointer” classes it was required to be parallel to the surface. Moreover, following each object relocation, the “open” motion was required to be triggered in either postures, depending upon the object being relocated (Fig. 3).

We propose a different explanation for this rather surprising finding; since acceleration is recorded on the skin surface, the associated measurement could be an alternative manifestation of the underlying muscular activity process that also gives rise to the electric field measured over the skin with EMG sensors. This may also be true for magnetometer data, which by measuring the magnetic field around the muscle area could indirectly provide an alternative measurement of muscular activity. The relationship between the two fields stems directly from the Maxwell-Ampère law which states that a changing electric field, due to muscle contraction in our case, generates a magnetic field. To validate this speculation, we ran the following experiment; we hypothesised that if such relationship exists between sEMG, accelerometer and magnetometer data, then it should be possible to use one type of signal to estimate another and vice-versa. We trained linear regression models to reconstruct the sEMG envelopes signals from IM data and found that the use of both accelerometer and magnetometer data yielded surprisingly accurate reconstructions of sEMG envelopes (Fig. 11). Certainly, there is no reason to expect that the relationship between the sEMG, accelerometer and magnetometer data should be linear and, therefore, one would expect to achieve higher decoding accuracies by using non-linear regression models. Nevertheless, the results from this experiment demonstrate that sEMG and inertial signals are indeed closely related, which provides evidence that they might reflect different and perhaps complementary aspects and impacts of the same underlying phenomenon, that is, the muscular activity. Consequently, it should come as no surprise that the combined sEMG-IM based decoder yields more accurate hand gesture recognition (Figs. 4, 7). The fact that gyroscope data alone failed in decoding both hand gesture and sEMG envelopes provides further support for this hypothesis. Taking everything into consideration, we argue that the added benefit of using IMs can be attributed to both their ability to capture dynamic spatial information, as well as to increase the robustness of muscular activity estimation which is subsequently employed to decode movement intention.

Another particular focus of our study was to investigate whether by combining multi-modal input data it would be possible to reduce the number of sensors required for real-time decoding without compromising performance. In accordance with previous studies which were limited to offline analyses [1, 5, 6, 35], we found that a few sensors only were required to virtually achieve the CA attained by the whole set of sensors (Figs. 4, 9). In the real-time experiment, however, we did observe a small decrease in performance for the able-bodied population (Fig. 7). As expected from the variability of myoelectric signals across subjects [41], the number of selected sensors varied amongst participants but was typically in the range of 4 to 6 (Fig. 10). A common observed pattern was the selection of sensors 1, 2, 3 and 10. The first three channels captured the activity of the extensor muscle group, while sensor 10 targeted the FDS muscle. For the amputee participant, the SFSS algorithm yielded three sensors only and, remarkably, the sEMG-IM subset condition (IV) achieved the best overall performance with 83% CR and an average CT of less than 40 s. To the best of our knowledge, efficient real-time prosthetic control by an amputee subject with as few as three sensors has not been previously reported.

This work is a proof of principle for integrating IMs in myoelectric control. Throughout our study, we used raw sensor values from IMUs which measured proper acceleration (accelerometer), angular velocity (gyroscope) and magnetic field (magnetometer). An alternative would be to perform sensor fusion and work with a different representation, such as quaternions or Euler angles [42]. Investigating the effect of different IM data representations in prosthetic control performance is subject of our current work.

## Conclusion

In this study, we demonstrated that the concurrent use of sEMG with IMs recordings, in conjunction with adopting an appropriate training data collection paradigm, can improve the performance of classification-based prosthetic hand control. We collected a large dataset comprising recordings with 22 subjects (20 able-bodied, two amputees) performing a range of 40 movements. We also conducted a real-time control experiment with 12 volunteers (11 able-bodied, one amputee) by using a state-of-the-art commercial prosthetic hand. Our results suggest that both offline classification accuracy as well as real-time performance can be improved when IMs are integrated in the decoding process. Finally, we found that by combining sEMG and IM data we were able to significantly reduce the number of sensors required to achieve top-level performance, a highly-desirable feature for clinical myoelectric applications.

## Endnotes

^1^ http://www.coaptengineering.com/

^2^ http://www.delsys.com/trigno-im/

^3^ Data collection with one of the amputee subjects was interrupted early due to a power supply failure and as a result, the participant did not perform the final two movements.

^4^ http://www.touchbionics.com/

^5^ http://www.shap.ecs.soton.ac.uk/

## Abbreviations

3D: Three-dimensional
AR: Auto-regressive
CA: Classification accuracy
CR: Completion rate
CT: Completion time
CV: Cross-validation
DOF: Degree-of-freedom
EDC: Extensor digitorum communis
EMG: Electromyogram
FDS: Flexor digitorum superficialis
IM: Inertial measurement
IMU: Inertial measurement unit
LDA: Linear discriminant analysis
LogVar: Log-variance
MAV: Mean absolute value
MLP: Multi-layer perceptron
MV: Mean value
RF: Random forest
RFN: Regulatory feedback network
ROS: Robot operating system
sEMG: Surface electromyography
SFSS: Sequential forward sensor selection
SVM: Support vector machine
TAC: Target achievement control
TD: Time-domain
WL: Waveform length
